# Can Exercise-Induced Muscle Damage Be a Good Model for the Investigation of the Anti-Inflammatory Properties of Diet in Humans?

**DOI:** 10.3390/biomedicines9010036

**Published:** 2021-01-05

**Authors:** Spyridon Methenitis, Ioanna Stergiou, Smaragdi Antonopoulou, Tzortzis Nomikos

**Affiliations:** 1 Sports Performance Laboratory, School of Physical Education & Sports Science, National and Kapodistrian University of Athens, 17237 Athens, Greece; smetheni@phed.uoa.gr; 2 Bioiatriki, Ergometric and Nutrition Center, 11526 Athens, Greece; joannste23@gmail.com; 3 Department of Nutrition & Dietetics, School of Health Sciences and Education, Harokopio University, 17671 Athens, Greece; antonop@hua.gr

**Keywords:** oxidative stress, experimental model, anti-inflammatory diets, inflammatory response, chronic inflammation, low grade chronic inflammation, inflammatory models

## Abstract

Subclinical, low-grade, inflammation is one of the main pathophysiological mechanisms underlying the majority of chronic and non-communicable diseases. Several methodological approaches have been applied for the assessment of the anti-inflammatory properties of nutrition, however, their impact in human body remains uncertain, because of the fact that the majority of the studies reporting anti-inflammatory effect of dietary patterns, have been performed under laboratory settings and/or in animal models. Thus, the extrapolation of these results to humans is risky. It is therefore obvious that the development of an inflammatory model in humans, by which we could induce inflammatory responses to humans in a regulated, specific, and non-harmful way, could greatly facilitate the estimation of the anti-inflammatory properties of diet in a more physiological way and mechanistically relevant way. We believe that exercise-induced muscle damage (EIMD) could serve as such a model, either in studies investigating the homeostatic responses of individuals under inflammatory stimuli or for the estimation of the anti-inflammatory or pro-inflammatory potential of dietary patterns, foods, supplements, nutrients, or phytochemicals. Thus, in this review we discuss the possibility of exercise-induced muscle damage being an inflammation model suitable for the assessment of the anti-inflammatory properties of diet in humans.

## 1. Introduction

Subclinical, low-grade, inflammation is one of the main pathophysiological mechanisms underlying the majority of chronic and non-communicable diseases [1,2,3,4,5]. It is therefore obvious that non-pharmacological interventions, such as dietary ones, aiming to modulate immune system, without compromising it, could serve as efficient ways of prevention while at the same time they could act complementarily to standard medication. In the last two decades, several dietary patterns different food items, phytochemicals, nutraceuticals, and supplements are promoted with the claim of possessing anti-inflammatory properties [6,7,8,9,10,11]. However, the impact of the above interventions in the immune system of humans remains uncertain since the majority of the studies have been performed under laboratory settings (e.g., cell culture and/or animal testing). Thus, the extrapolation of these results to humans is risky [12,13]. In addition, the majority of the clinical trials, exploring the “anti-inflammatory” effect of dietary/supplementation patterns on human subjects, assess their effectiveness based on their impact on a limited panel of inflammatory biochemical markers, mainly under controlled, fasted, non-stressed conditions. According to this, it will be of interest if we could assess the pro-inflammatory and/or anti-inflammatory profile of different dietary interventions, by developing inflammatory models, in humans, which could induce a transient inflammatory response to volunteers in a regulated and predicted fashion. The development of inflammatory conditions in humans, could be achieved, either by pharmaceutical or medical interventions, or by intentional injuries such as mechanical trauma, toxins, unhealthy lifestyles, and burns. Of course, the majority of these approaches have several ethical restraints that impair the development of such models. In contrast, ethical, controlled, and well-established models, to induce transient inflammation in humans, are certain exercise/training modalities, which are known to induce elevations of inflammatory-, oxidative stress-, and muscle-damage-related blood markers [14,15], impair clinical phenotypes, and alter metabolic procedures [16,17,18,19]. In this review article we discuss the possibility of exercise-induced muscle damage (EIMD) being a model of acute inflammation suitable for the assessment of the anti-inflammatory properties of diet in humans. 

## 2. The Protective Anti-Inflammatory Role of Nutrition in Chronic Diseases

The inflammatory responses are integral parts of the normal innate immune response conferring protection to infection and initiating mechanisms of repair and regeneration of damaged tissues [20,21]. Under acute inflammatory conditions, recognition receptors activate several signaling cascades, leading to the release of pro- and anti-inflammatory mediators, which orchestrate the recruitment of neutrophils and monocytes/macrophages to the damaged tissues while at the same time initiate the lysis of inflammation and the repair of tissue [20,21,22,23]. However, even a low to moderate chronic activation of inflammatory mechanisms induced either from long-term, persistent infections, autoimmune diseases, and/or increased daily inflammatory insults due to lifestyle (obesity, sedentary lifestyle, smoking, stress) and dietary habits (dense meals rich in simple sugars, trans fatty acids, advanced glycation end-products) overwhelms the anti-inflammatory processes of the immune system resulting in a chronic, sub-clinical inflammation [2,6,24,25,26,27,28,29,30]. The biochemical phenotype of this condition is mildly elevated levels of inflammatory mediators in the circulation. For example, C-reactive protein (CRP) levels are raised 2–3 fold under low-grade inflammation while those levels can be increased up to 10–1000 times in acute inflammation [31]. 

It is well documented that a subclinical activation of the inflammatory mechanisms may be a predisposing risk factor for non-communicable diseases such as cardiovascular disease, cancer, metabolic syndrome, diabetes, depression, dementia, and biological aging in general [1,2,3,4,25,32,33,34,35,36]. Actually, subclinical inflammation seems to underlie the link between unhealthy lifestyle with the pathogenesis of chronic diseases [2,23,29]. Taking into account the linear relationship between the levels of subclinical inflammation markers (e.g., CRP) and the risk for chronic diseases it is obvious that even a small attenuation of subclinical inflammation by lifestyle changes may confer protection against those diseases [7,36,37,38,39]. Therefore, dietary interventions targeting to reduce inflammation seem to be an efficient way of prevention for diseases with a chronic inflammatory background [7,9,10,30,40,41,42,43]. 

It is now known that prudent dietary patterns, such as the Mediterranean Diet [7,8,39,44,45] and macro/micro-nutrients, have a strong protective effect against non-communicable diseases, with a strong inflammatory profile [10,30,40,41,42,43]. For the majority of these studies, the assessment of the anti-inflammatory properties of diet was based on the measurement of few classical circulating markers (CRP, IL-6, TNFα). On the other hand, a plethora of microNutrients, phytochemicals, and supplements failed to attenuate inflammation in humans, despite their strong anti-inflammatory actions in cellular and animal studies [12,13,40,46]. The main reason for this discrepancy is the complexity of the pathophysiology of inflammation in humans which can be poorly replicated by animals or cell culture models. Taking into consideration the controversy between animal and human experimentation it seems that the development of more realistic, novel methodological tools for the study of inflammation directly to humans is highly important.

## 3. The Development of Inflammation Models in Humans Would Greatly Facilitate the Assessment of the Anti-Inflammatory Properties of Nutrition

Several methodological approaches have been applied for the assessment of the anti-inflammatory properties of nutrition. The majority of them is based on cellular and animal models of inflammation. Cellular models of inflammation include LPS-induced secretion of cytokines, expression of adhesion molecules in the surface of cells, phagocytosis activity, natural killer cells lysing capability against cancer cells etc. Cell-based assays were mainly utilized for the identification of the anti-inflammatory properties of isolated nutrients, extracts, and phytochemicals. Despite their usefulness for screening purposes and mechanistic studies the results of those studies cannot be extrapolated directly to humans [12,13,40,46]. The active dietary ingredients, in vivo, are found in much lower concentrations and in a more complex environment than those used in the cellular studies and in structural forms which may differ from the initial structures in foods due to in vivo metabolism [47].

The rapid growth of genetic engineering enabled the development of a plethora of animal models of inflammation by which the anti-inflammatory properties of diet could be assessed, in a more physiological way. Moreover, animal experiments provide wider access to the immune system (thymus, lymph nodes, bone marrow, peritoneal cavity). However, after many years of studying and working with animals in biomedical research, ethical issues have emerged concerning the reproducibility of animal models and their relevance with human inflammatory diseases [12,13,40,46,48]. The results from animal experiments cannot be easily extrapolated to humans because of the biological differences between species [12,13,40,46,48]. Over and above that, many studies in animal models are of poor methodological design exposing patients to unnecessary risk and wasting research funds. This is justified by the discrepancy between the outcomes of animal experiments and clinical trials [12,46,48].

The majority of human studies, investigating the association between diet and inflammation, are cross-sectional and prospective epidemiological studies [3,49,50,51,52,53,54]. The outcomes are based on the measurement of a small panel of soluble inflammatory mediators and hematological indices which by no way give a holistic view of the inflammatory system while at the same time is questionable whether the tools of the nutritional assessment are reliable to estimate dietary intakes accurately [3,49,50,51,52,53,54]. Randomized dietary interventions are the gold standard of human experimentation and several dietary interventions have been so far tried to assess the ability of dietary patterns, supplements, food items, and nutrients to modulate subclinical inflammation [3,49,50,51,52,53,54]. Although this is the best experimental approach so far, randomized dietary interventions lack mechanistic information since the interpretation of the results is based on the comparison of a limited panel of inflammatory indices, measured under static conditions in biological fluids, before and after the intervention. Even if a dietary intervention is able to favorably modulate inflammatory indices this could be the indirect result of diet on other risk factors of subclinical inflammation (e.g., weight loss) rather than a direct involvement to inflammatory mechanisms. In addition, most dietary intervention studies do not assess clinical phenotypes of inflammation either because they do not exist or they are difficult to be estimated in humans. Finally, most dietary intervention studies measure inflammatory mediators, at one-time point, under fasting and resting conditions which does not allow the assessment of intervention’s ability to modulate the response (plasticity) of the immune system under real inflammatory insults. It is therefore obvious that the development of an inflammatory model in humans, by which we could induce inflammatory responses to humans in a regulated, specific, and non-harmful way, could greatly facilitate the estimation of the anti-inflammatory properties of diet in a more physiological and mechanistically relevant way. We believe that exercise-induced muscle damage could serve as such a model, at least for acute/transient, self-limited inflammatory pathophysiological conditions. Considering this, several excellent studies, mainly coming from the field of sports nutrition, have already proven the ability of dietary interventions (protein supplements, phytochemicals, omega-3 fatty acids, BCAA) to diminish EIMD-induced inflammation, and thus to provide positive effects on muscle morphology/function, athletic performance and recovery, establishing the utility of EIMD as a suitable inflammatory model (for example: [5,14,15,55,56,57,58,59,60,61,62,63,64,65,66,67,68,69,70]).

## 4. Exercise-Induced Muscle Damage’s Prototypic Inflammatory Responses in Muscle Tissue

EIMD is a phenomenon that occurs either after a prolonged unaccustomed exercise, or after a very intense, high-demanding exercise (high intensity, long duration, high volume, high frequent, or combination of these), as a consequence of the very high mechanical and metabolic demands during the exercise [14,15,55,65,70,71,72,73,74,75]. EIMD is classified as a grade 1 muscular injury, characterized by minor ultrastructural muscle disruptions without a permanent defect, such as structural disruption of sarcomeres, disturbed excitation-contraction coupling and calcium signaling, and extended muscle protein degradation [14,15,55,71,72,73,74,75,76,77,78,79,80,81,82]. Although the pathology of EIMD is usually subclinical, the perceived sensation may vary from mild muscle stiffness to exhausting pain and a temporary reduction in both maximum strength and range of motion until several days after the stimuli [15,83,84]. It is also usually accompanied by sensitivity, swelling, or stiffness during palpation or motion of the damaged muscle, a process which is associated with delayed onset muscle soreness (DOMS) [73,85]. However, this inflammatory environment as a cellular response of muscle tissue damage is of high importance for the proper muscle tissue’s repair and regeneration [15,71,76,78,82,85].

The type of exercise and the type of muscle contractions applied are crucial determinants of the damaging and inflammatory responses. It is well established that the eccentric exercise, which involves only lengthening muscle actions, could lead to extensive EIMD and inflammation [15,55,72,74,84,86,87,88]. During eccentric exercises (isokinetic, downhill running, descending the stairs or a box) muscles are forced to lengthen, when the external forces (external weights, gravity, body weight) acting on them are greater than the forces that muscles can produce. This leads to an overstretching of sarcomeres, beyond their normal lengths [63,72,74,84,88,89,90,91,92,93,94,95,96,97,98,99]. During extensive sarcomere lengthening, there is a decreased overlapping of actin-myosin filaments, leading to a decreased number of active cross-bridge attachments, and thus to a decreased capability for active force generation [55,72,73,74,84,90,93,97,98,99,100,101,102,103]. In addition, although during eccentric contractions, muscles are capable to produce or absorb greater forces, as it reveals from the force–velocity curve [104], the motor units recruitment order is different between eccentric contractions and concentric, with type II muscle fibers to be recruited from the onset of muscle contraction even if the activation of muscle fibers is reduced compared to when the muscle performs maximal concentric contractions [105,106,107]. Thus, due to the fewer motor units that are recruited during eccentric contractions, as well as to the stretched and overstretched sarcomeres, where there is a reduced overlap of myosin and actin filaments, muscles are forced to overcome the external loads in a very adverse mechanical environment, in the same time that passive forces are dramatically increased leading to sarcomeres’ over-straining and thus a disruption of the sarcomere, probably due to titin-stretch-induced damage [74,84,90,97,98,99,102,108,109,110]. In addition, during repeated eccentric contractions, the weaker sarcomeres are the first ones that are affected by the above situations, by remaining over-stretched (probably due to the breakdown of titin) and becoming incapable to continue subsequent contractions [74,84,93,95,97,98,99,102,108,109,110]. Hence, the remaining, “stronger” sarcomeres receive even higher loads leading to over-exertion of them, too. This gradually leads to an extensive damage of sarcomeres, breakdown of sarcoplasmic reticulum membranes, and loss of calcium homeostasis in the myocyte [74,84,98,108,109,110]. At this point, the dramatic increase in cytoplasmic calcium causes activation of several calcium-dependent proteolytic and phospholipolytic processes along with disruption of myofibrillar proteins and sarcolemma of the damaged fibers [74,76,78,84,98,111,112,113,114,115,116,117,118]. In addition, eccentric exercise damages mitochondria, sarcoplasmic, and connective tissue network [55,84,87,98,116,117]. The damage appears to worsen within the days following eccentric exercise, reaching a peak at 24–72 h post exercise depending on the type, intensity and loads of exercise and then gradually disappears within 2 or 3 weeks after exercise [14,15,21,55,66,67,71,72,76,79,80,81,82,83,84,85,86,87,88,89,90,93,97,116,117]. 

**Figure 1:**
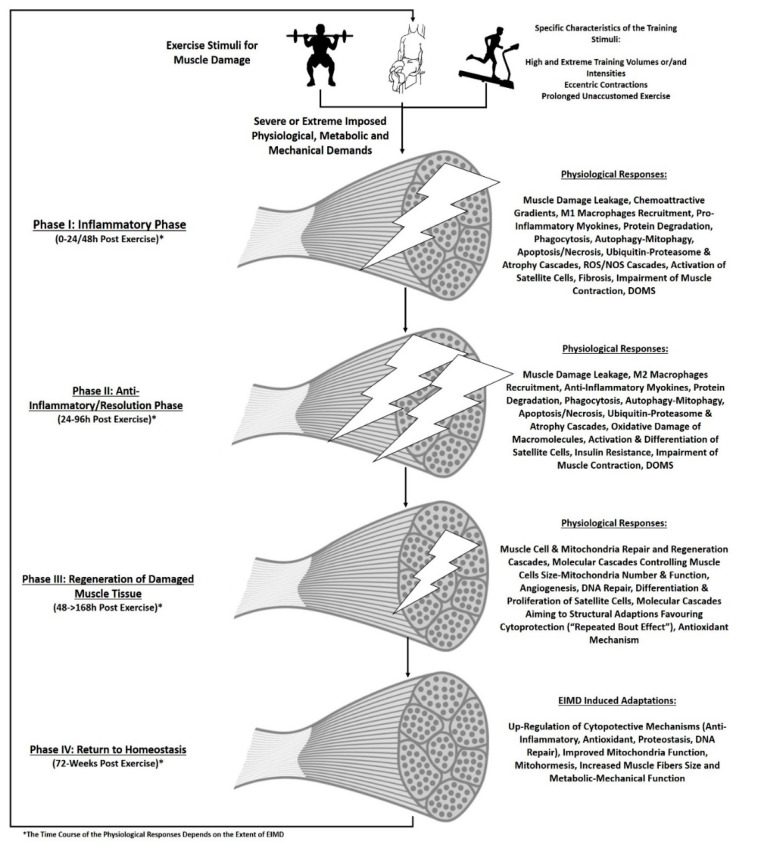
Time course of the physiological responses during exercise-induced muscle damage.

The inflammatory response, after EIMD, has been characterized quite well and several excellent original and review articles describe it in detail [15,21,71,74,76,77,78,79,90,93,119]. However, it should be mentioned that the majority of the mechanistic details presented in Figure 1 are based on cellular and animal studies while the inflammatory response in humans is not fully investigated and well addressed. Briefly, it begins immediately after the main mechanical damage when increased Ca^2+^ concentrations in the cytoplasm lead to degradation of muscle proteins and membrane phospholipids by activation of calpains and phospholipases A2 [114]. Meanwhile, three different types of inflammatory cells enter the injured area after muscle damage, namely, neutrophils and macrophages of type M1 and M2 [the analogs of rats’ CD68 (ED1+) and CD163 (ED2+) in humans].

Their main purpose is to degrade the damaged tissue by releasing active oxygen/nitrogen forms, and induce oxidative stress which is crucial for muscle regeneration after EIMD [14,71,76,87,93,117,120,121,122,123,124]. Neutrophils infiltrate the muscle tissue within a few hours and they phagocytose necrotic muscle fibers and cellular “remnant” while they constitute a source of pro-inflammatory myokines such as IL-1 and TNF-α [71,74,76,78,87,115,123,124,125,126]. Twenty-four hours post-EIMD macrophages appear in the damaged muscle tissue remaining for up to 14 days at the injured sites. The first subpopulation of macrophages are the macrophages expressing the ED1+ antigen and can enter into injured muscle fibers to phagocyte remnant cells and eroded myofibrils. The second subpopulation is ED2+ macrophages whose primary function is the secretion of growth factors and cytokines such as IGF-1, IL-6, and PDGF that can regulate proliferation and differentiation of myoblasts. Although, white blood cells are the main mediators of inflammation, fibroblasts and satellite cells are also “recruited” in the damaged area [71,115,127,128]. Fibroblasts have been shown to produce IL-6 and IL-1α, maintaining by this way the inflammatory response, and activating the proliferation of satellite cells to initiate the regeneration of damaged muscle fibers [71,125,128,129,130,131,132,133,134,135]. In addition, EIMD, elicits reactive oxygen and nitrogen species (RONS) which accompany the inflammatory response. According to the extent of muscle damage, RONS can further damage the muscle tissue but they also play a significant role for muscle regeneration and the adaptation of muscle tissues to eccentric exercise by mediating the up-regulation of antioxidant enzymes and the mitohormetic effects of exercise [14,16,18,70,79,120,136,137,138,139,140,141,142,143,144,145,146,147,148,149,150,151]. As a consequence of the activation of all the previous mentioned mechanisms in response to EIMD muscle cells’ autophagy, apoptosis, and regeneration-adaptation molecular mechanisms are upregulated, to reinforce the regeneration of muscle cells [103,136,141,144,146,148,152,153,154,155,156,157,158,159,160,161,162,163,164,165,166,167,168,169,170,171,172,173,174,175,176,177,178,179,180,181,182]. Thus, EIMD is also a successful model to investigate the effect of dietary compounds on the stress-induced mechanisms of autophagy, apoptosis, and regeneration-adaptation molecular cascades. Table 1 summarizes the most common clinical and biochemical indices that are affected by EIMD.

## 5. Exercise-Induced Muscle Damage and Its Methodological Advantages as an inflammatory Model in Humans

**Figure 2:**
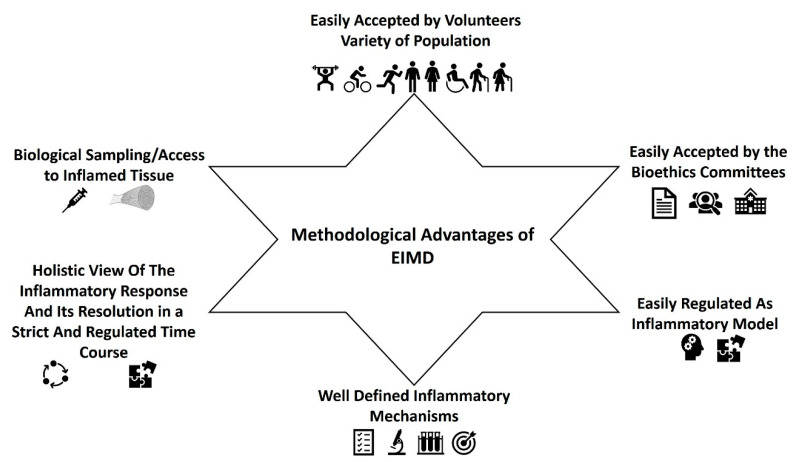
Methodological advantages of exercise-induced muscle damage.

In our opinion, EIMD is a convenient, dynamic, and informative model of inflammation. It can be applied either in studies investigating the homeostatic responses of individuals under inflammatory stimuli or for the estimation of the anti-inflammatory or pro-inflammatory potential of dietary patterns, foods, supplements, nutrients or phytochemicals. The main advantages of EIMD are the following (Figure 2):

- EIMD can be easily accepted by the volunteers and the bioethics committees. It can be easily induced by different types of exercise/training either in the lower or the upper limbs (see below). The inflammatory response can be applied to all kinds of populations irrespective of their health and training status, age, gender, race, body composition etc. Most importantly, volunteers easily consent to this kind of intervention which is actually just about exercise for them. In addition, most bioethics committees would have no objections to this kind of experimentation in humans.
- EIMD can be easily applied to humans in a regulated manner. Exercise scientists can induce muscle damage by forcing muscles to lengthen while generating active tension, with various stimuli. As it has been discussed in the previous section, EIMD can be easily induced through eccentric training after 85 to 300 maximal eccentric contractions, while the magnitude and the extent of EIMD from eccentric exercise, seems to be higher and prolonged (lasting for 72 h until 1–2weeks) compared to other type of exercises [14,15,55,62,63,64,66,67,70,72,74,79,84,86,88,90,93,94,97,112,113,117,122,132,183,184,185,186,187,188,189,190,191,192,193,194,195,196,197,198,199,200]. It should be mentioned that eccentric EIMD can be used and recommended for the prevention and/or rehabilitation of many chronic health conditions [94,188,199,201,202,203,204,205,206,207,208,209,210,211,212,213,214,215,216,217,218,219,220,221] since eccentric exercise has about 2–4 times lower metabolic and cardiovascular demands compared to other types of exercises [96,222]. Therefore, it seems that the use of eccentric exercise is a safe, effective, and regulated way to induce EIMD in all population groups. However, other training stimuli could also be applied. For example, high volume drop jump sessions (≥100 jumps) [223,224,225,226,227,228], or in general exercises with an increased volume of stretch-lengthening cycle movements [229], prolonged moderate to high intensity running [196,230,231], cycling [232,233,234,235], and downhill running [236,237,238,239,240], have been repeatedly reported to induce significant EIMD, lasting for more than 72–96 h post-training. Although traditional resistance training (e.g., 60–80% of 1RM, 4–8 sets per exercise) can induce also EIMD [241,242,243] and trigger immune [244,245,246,247,248,249] and inflammation-related molecules (e.g., cytokines and chemokines) responses [245,246,247,248,249,250,251,252], the extent of EIMD is limited or significantly lower and with shorter duration than the above type of exercises, especially compared to eccentric training [74,117,202,253,254]. For most of these exercises, no special instrumentation is required. Independent of the exercise type, EIMD will be stronger and longer if the exercise used is characterized by very high mechanical and metabolic demands, e.g., with high volumes and intensities, fast contraction velocities, high contraction frequencies, short rest periods, and at long muscle lengths [14,15,55,62,63,64,65,66,67,70,71,72,73,74,75,79,84,86,88,90,93,97,112,113,117,122,132,183,184,185,186,187,188,189,190,191,192,193,194,195,196,197,198,199,200,201,202,203,204,205,206,207,208,209,210,211,212,213,214,215,216,217,218,219,220,221,222,223,224,225,226,227,228]. However, it seems that EIMD is even greater and with longer duration in untrained participants [255] and when a new/different training stimuli (unaccustomed exercise) that voluntaries are not familiar with it is applied [256].
- The inflammatory mechanisms underlying EIMD are well defined. The muscle microtrauma, induced by the different types of exercise, but mostly from eccentric exercise, can trigger a typical cascade of inflammatory events that resemble aseptic inflammation after tissue damage (see above). It is therefore easier for researchers to identify the crucial mechanistic points that each intervention could affect.
- One of the biggest advantages of EIMD, is that researchers could have the whole picture of the inflammatory response and its lysis in a strict and regulated time course. In contrast, when individuals with already established low-grade, chronic inflammation are recruited, the variability of the clinical and biochemical phenotypes, pharmacology, and medical history is usually large even in well-controlled studies. Thus, even in the best controlled cross sectional studies, the diversity between the participants would have a strong conflicting impact on research outcomes.
- Biological sampling. Apart from the classical blood or saliva samples, that are usually collected before and several time points after the exercise trial this type of experiments allow you to take samples of the inflamed tissue, namely muscle biopsies. This technique has been used in many studies, investigating either the training-induced adaptations on muscle fibers (for example [89,122,257,258,259,260,261,262,263]), or muscle damage-inflammation (for example [63,122,128,231,248,264,265,266,267,268,269,270,271]. Muscle samples for such type of studies are usually obtained with Bergstrom needles from vastus lateralis of lower extremities, under local anesthesia, easy and quite safe for the volunteers, while in the majority of the countries, this is well accepted from the bioethics committees. The main advantage of taking muscle samples is that researchers, can investigate inflammation straight on the inflamed tissue and its cells in contrast to the majority of the studies where the inflammatory mechanisms are inferred by the alteration of biochemical markers in the circulation. Muscle biopsies can provide important information on the extent of sarcomere damage, of intra-cell biochemical-molecular procedures and/or genetic background of EIMD.
- The kinetics of clinical phenotypes linked to the inflammatory response can be easily determined. Such phenotypes are delayed-onset muscle soreness, maximum isometric torque, range of motion, limb circumference, and several other types of ergometric tests according to the inflamed limb.

## 6. Applications of Exercise Induced Muscle Damage as a Model of Inflammation 

The EIMD model can serve as a precise model, in studies investigating the effects of nutritional and training interventions, on acute and chronic inflammation conditions, mainly in metabolic-related inflammatory conditions. As for example, EIMD could serve as a useful model in human studies investigating:

- Acute and chronic inflammations. EIMD inflammatory response share similar pathophysiological and biochemical responses with acute and chronic inflammation. Thus, EIMD could find application in studies investigating the effect of nutrition and/or exercise, in acute and chronic inflammatory conditions such as those observed before and/or during the majority of chronic and non-communicable diseases [1,2,4,5,8,9,20,24,29,32,33,34,128,272]. For example, the inflammatory status of certain population groups (e.g., obese vs. normal weight) can be better assessed and compared under the dynamic conditions of EIMD. Taking into account that muscle biopsies can also be obtained and then the molecular mechanisms of autophagy, apoptosis and regeneration-adaptation could also be studied [75,103,136,141,144,146,148,152,153,154,155,156,157,158,159,160,161,162,163,164,165,166,167,168,169,170,171,172,173,174,175,176,177,178,179,180,181,182].
- Considering the pathophysiology behind conditions such as muscle/neurogenic inflammation, atrophy, cachexia, sarcopenia, and chronic muscle protein degradation EIMD can serve as a very reliable and regulated model to investigate how nutrition and or exercise may affect the physiological and biochemical background of those conditions.
- Ischemic preconditioning (IPC). After an EIMD stimuli, the following exercise bouts induce lower muscle damage and inflammation, due to the specific muscle adaptations, that minimize the extent of muscle damage, a phenomenon that it is known as “repeated bout effect” (RBE [79,84,273,274,275,276]). IPC protective mechanisms are comparable to those of RBE. IPC attained after one to five cycles of intermittent bouts of Ischemia/reperfusion, provide protection against the possibility of subsequent ischemia with longer duration. It has been documented that ROS are the main cardioprotective factor of IPC. Just a single bout of IPC produces a significant amount of ROS from mitochondria which trigger the protective signaling cascade [139]. Almost the same mechanisms seem to induce the RBE after repeated bouts of training sessions [79,84,273,274,275,276], providing further support that EIMD is a very good model to investigate the IPC and the hormetic effects of diet on those mechanisms.
- Rhabdomyolysis. Rhabdomyolysis is a pathophysiological condition of extensive skeletal muscle cell damage which could be induced from many physical (trauma, strenuous muscle exercise, electrical current) and non-physical causes (metabolic syndrome, drugs, electrolyte imbalance) [277]. This is a frequent phenomenon in patients taking statins, which may lead to unfavorable effects ranging from myalgia or myopathy to rhabdomyolysis and sometimes to acute renal failure [277]. The initial metabolic hypothesis of statin myopathy is that mitochondrial function is reduced while neutral lipids are increased, and ubiquitin proteasome is activated. In this case the activation of ubiquitin induces acceleration of proteolysis leading to muscle break down, atrophy, and necrosis [278]. Again, drug- and non-drug-induced rhabdomyolysis, have the same mechanisms and responses as those founded during an EIMD situation, specifically of those that are observed during the first 24–48 h post-exercise [14,15,18,21,55,63,65,66,71,72,74,75,76,77,78,79,80,87,90,93,103,116,117,120,121,122,123,136,137,138,140,141,142,143,144,146,148,152,153,154,155,156,157,158,159,160,161,162,163,164,165,166,167,168,169,170,171,172,173,174,175,176,177,178,179,180,181,182,196,197,198,200,279,280,281,282,283,284,285]. Therefore, EIMD can be applied in studies investigating the phenotypes that are more prone to rhabdomyolysis or in studies investigating the protective effects of dietary compounds to it.
- Fibromyalgia (FM). FM is a complex syndrome characterized by widespread pain that affects many tissues and the presence of allodynia and hyperalgesia [286,287]. Fatigue and functional disorders usually appear during the syndrome [286,287]. Pain is a common feature of EIMD and the physiological-metabolic mechanisms-responses observed in FM are similar to those found during EIMD [288].

## 7. Conclusions 

According to the above, it seems that EIMD is a controlled, highly regulated tool in the hands of researchers, to investigate the ability of different types of nutritional and training interventions to interfere with the progression and lysis of acute inflammatory conditions. It is worth testing if the ability of those interventions to attenuate acute inflammatory responses after EIMD can predict their ability to also act protectively against chronic inflammation although such as link has not been established yet. Nevertheless, EIMD allows the assessment of the putative anti-inflammatory, hermetic, or immunomodulatory properties of dietary intervention directly to human beings under real, dynamic conditions.

## Figures and Tables

**Table 1:** Clinical and biochemical indices affected by the inflammatory response after exercise-induced muscle damage.

Physiological Response	Marker
Pain/Delayed Muscle Soreness	Perceived Muscle Soreness by Visual Analog Scale or Algometers
Muscle Function	Rate of Force/Torque Development, Maximum Strength Power, Range of Motion, Muscular Work
Oedema	Limb Circumferences
Oxidative Stress	Protein Carbonyls, MDA, Isoprostanes, GSH/GSSG, Antioxidant Enzymes (Glutathione Peroxidases, Superoxide Dismutase, Catalase), Total Antioxidant Capacity, Antioxidant Vitamins
Muscle Damage	CK, LDH, Myoglobin, T-Troponin, Hydroxyproline, Hydroxylysine
Systemic Inflammation	WBC Count, IL-6, TNF-a, CRP, IL-10, IL-8, IL-1Ra, Lipid Mediators, INF-γ, MCP1, MIP-1
